# Antibody response to 13-valent pneumococcal conjugate vaccine is not impaired in patients with rheumatoid arthritis or primary Sjögren’s syndrome without disease modifying treatment

**DOI:** 10.1186/s41927-018-0019-6

**Published:** 2018-04-05

**Authors:** Per Nived, Tore Saxne, Pierre Geborek, Thomas Mandl, Lillemor Skattum, Meliha C. Kapetanovic

**Affiliations:** 1 Department of Clinical Sciences Lund, Section of Rheumatology, Lund University, Skåne University Hospital, SE-221 85 Lund, Sweden; 2 Department of Infectious Diseases, Central Hospital Kristianstad, J A Hedlunds väg 5, SE-291 85 Kristianstad, Sweden; 3 Department of Clinical Sciences Malmö, Section of Rheumatology, Lund University, Skåne University Hospital, Malmö, Sweden; 4 Department of Laboratory Medicine, Section of Microbiology, Immunology and Glycobiology, Lund University, Lund, Sweden; 5 Clinical Immunology and Transfusion Medicine, Lund, Region Skåne Sweden

**Keywords:** Rheumatoid arthritis, Sjögren’s syndrome, Pneumococcal vaccination, Pneumococcal conjugate vaccine

## Abstract

**Background:**

Pneumococcal vaccination is recommended to patients with rheumatoid arthritis (RA) and primary Sjögren’s syndrome (pSS). However, little is known whether the diseases influence pneumococcal vaccine response. This study aimed to investigate antibody response and functionality of antibodies following immunization with 13-valent pneumococcal conjugate vaccine (PCV13) in RA patients or pSS patients without disease modifying anti-rheumatic drugs (DMARD), compared to patients with RA treated with DMARD or to healthy controls.

**Methods:**

Sixty RA patients (50 without DMARD and 10 with MTX), 15 patients with pSS and 49 controls received one dose of PCV13. Serotype-specific antibody concentrations for pneumococcal polysaccharides 6B and 23F and functionality of antibodies (23F) were determined in serum taken before and 4–6 weeks after vaccination using ELISA and opsonophagocytic activity assay (OPA), respectively. Proportions of individuals with positive antibody response (i.e. ≥ 2-fold increase from prevaccination concentrations; antibody response ratio; ARR ≥ 2), percentage of individuals reaching putative protective antibody level (i.e. ≥1.3 μg/mL) for both serotypes, and difference in OPA were calculated.

**Results:**

After vaccination, antibody concentrations for both serotypes increased in RA without DMARD (*p* < 0.001), pSS (*p* ≤ 0.05 and < 0.01) and controls (*p* < 0.001). Antibody responses to 6B and 23F were comparable in RA without DMARD (64% and 74%), pSS (67% and 53%) and controls (65% and 67%), but lower in the small group RA with MTX (both 20%, *p* < 0.01). Similarly, significant increases of patients reaching protective antibody levels were seen in RA without DMARD (*p* ≤ 0.001) and controls (*p* < 0.001). After vaccination, OPA increased significantly in controls, RA and pSS without DMARD (*p* < 0.001 to 0.03), but not in RA with MTX.

**Conclusions:**

Pneumococcal conjugate vaccine is immunogenic in RA and pSS patients without DMARD and in line with previous studies we support the recommendation that vaccination of RA patients should be performed before the initiation of MTX.

**Trial registration:**

ClinicalTrials.gov Identifier: NCT02240888. Retrospectively registered 4 September, 2014.

## Background

Infection, in particular pneumonia, is an important cause of the excess mortality in patients with rheumatoid arthritis (RA) [1] and recurrent pulmonary infections are reported in 10–35% of Sjögren’s syndrome patients [2]. Doran et al. found an 80% increased risk of infection in a retrospective cohort of RA patients, compared to age- and sex-matched subjects without RA [3]. In addition, the authors reported that older age, presence of extra-articular manifestations, leukopenia, comorbid conditions (e.g. diabetes mellitus), and use of corticosteroids, but not traditional disease modifying anti-rheumatic drugs (DMARDs), were strong predictors of infection [4]. More recently, a systematic review and meta-analysis by Singh et al. showed that in comparison to traditional DMARDs, standard-dose biological drugs and high-dose biological drugs were associated with an increased risk of serious infections, (Odds ratios 1.31 and 1.90, respectively) [5].

Invasive pneumococcal disease (IPD), caused by *Streptococcus pneumoniae*, is a vaccine-preventable life-threatening condition. Retrospective cohort studies have demonstrated increased risks of IPD in patients with autoimmune inflammatory rheumatic diseases, i.e. RA (Rate ratio [RR] 2.47, 95% confidence interval [CI] 2.41–2.52) and primary Sjögren’s syndrome (pSS) (RR 3.2, 95% CI 2.9–3.5) [6].

Two pneumococcal vaccine types are currently available for adults, the 23-valent pneumococcal polysaccharide (PPV23) and the 13-valent conjugate vaccine (PCV13). The advantage with the conjugate vaccine is the T cell-dependent (TD) immune response, resulting in the production of memory B-cells. Our group have previously reported that antibody responses to PPV23 [7] and 7-valent pneumococcal conjugate vaccine (PCV7) [8] are impaired in chronic arthritis patients during treatment with methotrexate (MTX), but not with tumour necrosis factor (TNF) inhibitor monotherapy. The current recommendation for adults with immunocompromising conditions, is to receive immunization with a dose of PCV13, followed in at least 8 weeks by a dose of PPV23 [9]. RA and pSS are systemic autoimmune inflammatory diseases, but little is known whether the immunological disturbance as a part of disease itself influences the immune response to PCV13 vaccination since studies of the vaccine response in untreated RA and pSS patients are scarse or non-existing. The aim of this study was to investigate if antibody response and functionality of antibodies following immunization with PCV13 is impaired in patients with RA and pSS without active anti-rheumatic treatment compared to RA patients treated with methotrexate (MTX), or to healthy controls.

## Methods

### Patient inclusion

Adult patients with RA and pSS, regularly monitored at the Department of Rheumatology in Lund and Malmö at Skåne University Hospital were eligible for this study. At inclusion in the present study all patients’ medical records were scrutinised in order to confirm that patients fulfilled the American College of Rheumatology (ACR)/ European League Against Rheumatism (EULAR) criteria for RA or pSS [10, 11]. Ongoing treatment at the time of vaccination was noted as a basis for later patient stratification. Patients were excluded from the study if anti-rheumatic treatment had been changed within 4 weeks before vaccination, if they had been previously vaccinated with PPV23 within 1 year, had a history of allergic reaction at previous vaccinations, were pregnant, or had an ongoing infection. Healthy control subjects were recruited from the staff and relatives at the department of Rheumatology in Lund. The controls were younger (median 57.2 years) than RA patients (median 66.9 years, *p* < 0.001).

### Ethics approval and consent to participate

The study was approved by the Regional Ethical Review Board at Lund University, Sweden (Dnr 2011/341). Consecutive patients fulfilling inclusion criteria were invited to participate in the study. All invited participants were provided with oral and written information, and written consent was obtained before study enrolment.

### Vaccination protocol

All participants received a single 0.5 mL dose of PCV13 (Prevenar 13®, Pfizer) administered as an intramuscular injection in the deltoid muscle. At the time of vaccination, a rheumatologist performed a clinical examination and data were collected on disease and treatment characteristics and previous vaccinations using a structured protocol. All participants were encouraged to report any adverse events following vaccination, as well as possible changes in rheumatic disease. Adverse events (AEs) and adverse drug reactions (ADRs) were recorded in line with the Guideline for Good Clinical Practice and Clinical Safety Data Management [12].

### Pneumococcal serotype-specific IgG measurement

Serum samples were collected immediately before and 4–6 weeks after vaccination. Serotype-specific IgG antibody concentrations for pneumococcal serotypes 6B and 23F, both included in PCV13, were quantified using enzyme-linked immunosorbent assay (ELISA). The World Health Organization consensus pneumococcal IgG ELISA [13] was performed with minor modifications, described previously [14].

### Opsonophagocytic activity (OPA) assay

Flow cytometric OPA assay was performed for pneumococcal serotype 23F. The original method has been described by *Martinez* et al. [15] and it was executed with some modifications [14].

### Statistical analysis

Differences between groups were analysed using the Chi-square test and the Mann-Whitney U test when appropriate. Geometric mean Ab concentrations (GMCs) and 95% confidence intervals (95% CI) were calculated from log-transformed values. Pre- and post-PCV13 GMCs for larger groups (*n* > 30) were compared using paired samples t-test. Pre- to postvaccine OPA and antibody changes in smaller groups were compared using Wilcoxon signed rank test. Differences in GMCs between patients (*n* > 20) and controls were analysed using independent samples t-test. A positive antibody response was defined as an antibody response ratio (ARR, i.e., the ratio of post- to prevaccination antibody levels) ≥2. Putative protective level for each serotype was defined as a titer ≥1.3 μg/mL, as recommended by the American Academy of Allergy, Asthma & Immunology [16]. Predictors of positive antibody response for both serotypes were analysed using logistic regression model, adjusted for age. Correlations between “% changes in OPA” and ELISA difference in each groups were calculated using Spearman’s rank correlation test. Statistical calculations were performed using IBM® SPSS® version 23 and diagrams were drawn using Graphpad Prism® version 6.

## Results

A total of 60 patients with RA (50 without DMARD and 10 on MTX), 15 patients with pSS without DMARD and 49 controls were vaccinated. None was lost to follow up. In the RA without DMARD group 58% were treated with prednisolone (median dose 5 mg daily, range 0–15 mg). The demographic details, disease characteristics, pre- and postvaccination geometric mean antibody concentrations of the participants are shown in Table 1. Prevaccination GMCs neither differed significantly between the patient groups nor compared to controls. Pre- to postvaccination increases of serotypes 6B and 23F GMCs were found in patients with RA without DMARD (both *p* < 0.001), pSS (*p* = 0.05 and *p* = 0.006) and controls (both *p* < 0.001). The small patient group RA with MTX only showed increase in serotype 6B GMC (*p* = 0.05).

**Table 1:** Demographic and patient characteristics in treatment groups and controls

	RA without DMARD (*n =* 50)	RA with MTX (*n =* 10)	pSS without DMARD (*n* = 15)	Controls (*n =* 49)
Age, median (range) years	66.9 (35–87)	67.4 (39–79)	62.3 (25–89)	57.2 (17–85)
Sex (% female)	78.0	70.0	87.0	63.3
Disease duration, mean (range) years	5.6 (0–36)	13.1 (2–40)	7.0 (0–23)	–
CRP, median (range) mg/L	7 (0–78)	3 (0–11)	1.7 (0.7–38)	–
ESR, median (range) mm/h	21 (4–71)	16 (5–42)	12 (7–66)	–
RF positive, %	78	80	43	–
ACPA positive, %	69	70	8	–
ANA positive, %	–	–	73	–
Anti-ENA positive, %	–	–	73	–
Anti-SSA (anti-Ro) positive, %	–	–	67	–
Anti-SSB (anti-La) positive, %	–	–	40	–
Prednisolone, %	58	0	13	0
Dose, median (range) mg/day	5 (0–15)	0	0 (0–10)	–
Previous PPV23, *n* (%):	1 (2)	0	0	3 (6)

*DMARD* Disease modifying anti rheumatic drugs, *RA* Rheumatoid arthritis, *pSS* primary Sjögren’s syndrome, *CRP* C-reactive protein, *ESR* Erythrocyte sedimentation rate, *ACPA* Antibodies against citrullinated peptides, *ANA* Antinuclear antibodies, *Anti-ENA* Antibodies against extractable nuclear antigens, *Anti-SSA (anti-Ro)* Anti-Sjögren’s-syndrome-related antigen A, *Anti-SSB (anti-La)* Anti-Sjögren’s-syndrome-related antigen B, *RF* Rheumatoid factor

### Antibody response

**Fig. 1:**
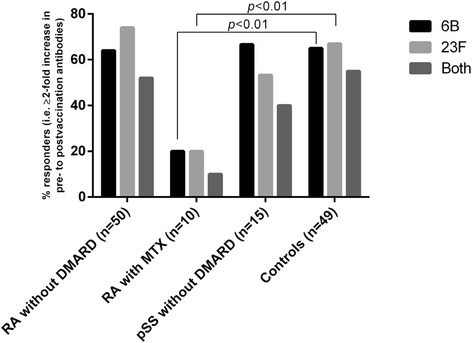
Proportions of subjects with a positive antibody response PCV13 vaccination

Proportions of subjects with a positive antibody response (i.e. antibody response ratio; ARR ≥ 2) to serotypes 6B and 23F was decreased in patients with RA on MTX treatment (both *p* < 0.01), but not in RA without DMARD or pSS without DMARD, compared to controls (Fig. 1). Proportions of antibody responders to both serotypes did not differ significantly between groups RA without DMARD (52%), pSS without DMARD (40%) and controls (55%). When a logistic regression analysis was performed, RA on MTX was the only group with a significant lower positive antibody response to both serotypes. Due to limited number of patients in this group and patients with pSS only adjustment for age could be performed (Table 2).

**Table 2:** Predictors of a positive antibody response (i.e. antibody response ratio; ARR ≥ 2) to both 6B and 23F

	*p*-value	OR	95% CI
RA without DMARD (yes/no)	0.161	1	
RA with MTX (yes/no)	0.037	0.10	0.01–0.87
pSS without DMARDs (yes/no)	0.370	0.58	0.18–1.90
Controls (yes/no)	0.992	0.99	0.42–2.39
Age at vaccination (years)	0.496	0.99	0.97–1.02

### Putative protective levels

The proportions of subjects reaching above protective antibody level (i.e. ≥1.3 μg/mL) for serotype 6B increased after vaccination in RA without DMARD (*p* = 0.001) and controls (*p* < 0.001) but not in RA with MTX and pSS without DMARD (Table 3). For 23F, increases were found in RA without DMARD (*p* < 0.001), pSS without DMARD (*p* = 0.05) and controls (*p* < 0.001) but not in RA with MTX. The pre- to postvaccination percentage increase for serotype 6B and 23F respectively were 24% and 42% in RA without DMARD, 10% and 20% in RA with MTX, 14% and 26% in pSS without DMARD, and 28% and 40% in controls.

**Table 3:** ELISA IgG geometric mean concentrations and proportions of subjects reaching above putative protective antibody concentration (i.e. ≥1.3 μg/mL) before and after vaccination in treatment groups and controls

	RA without DMARD (*n =* 50)	RA with MTX (*n =* 10)	pSS without DMARD (*n* = 15)	Controls (*n =* 49)
Geometric mean concentrations (μg/mL):				
6B prevaccination (95% CI)	0.6 (0.3–1.0)	1.3 (0.5–2.9)	0.6 (0.2–2.0)	0.8 (0.5–1.3)
6B postvaccination (95% CI)	3.4 (1.8–6.0)	2.1 (0.8–5.6)	2.3 (0.8–6.5)	3.1 (1.9–5.0)
*P* of increase	< 0.001	0.05	0.05	< 0.001
23F prevaccination (95% CI)	0.5 (0.3–0.8)	1.0 (0.3–3.5)	0.9 (0.4–1.9)	0.6 (0.4–0.9)
23F postvaccination (95% CI)	2.5 (1.5–4.1)	1.7 (0.7–3.9)	3.5 (1.1–11.5)	3.3 (2.0–5.5)
*P* of increase	< 0.001	0.39	0.006	< 0.001
Proportions of subjects with IgG concentration ≥ 1.3 μg/mL (%):				
6B prevaccination (95% CI)	38 (24–52)	60 (23–97)	33 (6–60)	37 (23–51)
6B postvaccination (95% CI)	62 (48–76)	70 (35–100)	47 (18–75)	65 (51–79)
*P* of increase	0.001	0.32	0.16	< 0.001
23F prevaccination (95% CI)	26 (13–39)	40 (3–77)	47 (18–75)	29 (15–42)
23F postvaccination (95% CI)	68 (55–81)	60 (23–97)	73 (48–99)	69 (56–83)
*P* of increase	< 0.001	0.16	0.046	< 0.001

### Opsonophagocytic activity

**Fig. 2:**
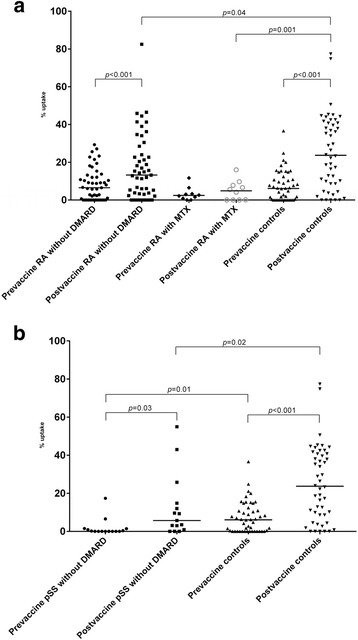
**a** Opsonophagocytic activity before and after PCV13 vaccination in RA and controls. **b** Opsonophagocytic activity before and after PCV13 vaccination in primary Sjögren’s syndrome and controls

After vaccination, OPA increased in groups RA without DMARD (*p* < 0.001), pSS without DMARD (*p* = 0.03) and controls (*p* < 0.001) but did not change significantly in patients with RA on MTX. The mean percentage change in OPA was lower in RA on MTX (1.8%, *p* = 0.01), RA without DMARD (8.9%, *p* < 0.01), but not in pSS without DMARD (10.5%), compared to controls (17.9%). Pre- and postvaccination OPA in patients with RA and pSS is shown in Fig. 2a and b, respectively. Positive correlations between percentage change in OPA and pre- to postvaccination increase in ELISA were found for patients with RA without DMARD (ρ = 0.28, *p* = 0.03) and controls (ρ = 0.45, *p* = 0.001).

### Effects of age and glucocorticoid treatment

Neither antibody response ratio nor pre- to postvaccination increase in ELISA correlated with age for all patients, subgroups, or controls. A negative correlation was found between percentage change in OPA and age in controls (ρ = − 0.36, *p* = 0.01) but not in patients. Prednisolone dose did not correlate with antibody response or percentage change in OPA.

### Vaccine safety

The vaccine was generally well tolerated, all reported side effects were considered mild-moderate and temporary (duration 1–2 days up to 1–2 weeks). The following side effects were reported in the group RA without DMARD: pain/skin reaction around injection site (*n* = 5), increased arthralgia (*n* = 3), upper respiratory tract symptoms (*n* = 2) and nausea (*n* = 1). In the group RA with MTX, 7 patients had low disease activity and 3 patients no activity (DAS28) at the time of vaccination. None of the patients reported increased disease activity or relapse after vaccination.

## Discussion

This study demonstrated that pneumococcal conjugate vaccine was immunogenic in patients with RA and pSS syndrome without DMARD treatment, and the antibody response rates were comparable to those of healthy controls. In contrast, a small group of RA patients with MTX treatment had lower antibody responses compared to controls, in accordance with previous findings of our group [8]. These results are in accordance with a meta-analysis of studies investigating immunogenicity of pneumococcal vaccine in RA finding that MTX treated patients with RA had decreased ability to respond to pneumococcal polysaccharide vaccine [17]. Functionality of antibodies after vaccination, measured by an opsonophagocytosis assay, was lower in RA patients without DMARD compared to controls, and a weak positive correlation was found between OPA and ELISA in this group. However, we found a negative correlation between percentage change in OPA and age in controls, and the fact that controls were younger than RA patients could contribute to the difference in functional immune response. To the authors’ knowledge, this is the first study of the immune response to pneumococcal conjugate vaccine in patients with RA and pSS without DMARD treatment. The underlying autoimmune pathologies of these diseases are well known to increase the risk of serious infections [3, 6, 18], and pneumococcal vaccination is important to prevent invasive disease. Our findings, i.e. that pneumococcal conjugate vaccine was immunogenic in both RA and pSS without DMARD, support the use of this vaccination in newly diagnosed patients before starting immunosuppressive DMARD treatment.

Among the strengths of this study were the use of both ELISA and a functional antibody assay to evaluate the immune responses. No patients were lost to follow up.

Our study had some limitations. First, we only performed ELISA for two serotypes (6B and 23F). Although the serotypes are among the most common causes of IPD in our part of Sweden [19], we cannot be certain that they are representative of the immune response to PCV13. At the same time, the studies on the immunogenicity of other pneumococcal serotypes prior to the licensure of the conjugate vaccine did not reveal any significant differences between serotypes and probably the impact of the rheumatic diseases or their treatment on antibody response would be similar for the other serotypes [20]. Second, functionality of antibodies was evaluated with a phagocytic OPA assay to only one serotype (i.e. 23F), although validated previously [15], it contrasts to the killing-type OPA assay which is now considered the standard method for vaccine evaluations [21]. Further, small sample sizes may limit the generalizability of our results. Although the group of RA patients with MTX treatment (*n* = 10) arguably is too small for statistical analyses, the purpose of this group was merely to illustrate and confirm what previous studies have shown, i.e. that methotrexate decreases the antibody response to pneumococcal vaccination. The seemingly higher prevaccination antibody level of 6B in this group is probably a result of prior infections and it can explain the lower antibody response to this serotype. The fact that controls were younger than RA patients was considered a minor limitation, and it doesn’t contradict our main findings, i.e. that antibody responses in patients with RA without DMARD were comparable to those of healthy controls.

In a recent study, adjuvanted H1N1 influenza vaccination of untreated pSS patients resulted in higher specific IgG titers compared to healthy controls, and autoantibody (Ro/SSA and La/SSB) titers increased. Endosomal toll-like receptor stimulated naïve B-cells in vitro were shown to differentiate and class switch more readily from untreated pSS patients compared with patients receiving hydroxychloroquine and healthy controls [22]. However, in our study with pneumococcal vaccine, this enhanced antibody response could not be reproduced, which could possibly be due to the fact that not all pSS patients in the current study were anti-Ro/SSA and La/SSB seropositives.

## Conclusions

Antigen challenge using pneumococcal conjugate vaccine in patients with RA or pSS without active anti-rheumatic treatment resulted in antibody response comparable to that of healthy controls. Similar to previous findings a decreased response to this vaccine was observed in RA patients treated with methotrexate.

These findings are in line with the previous recommendation that pneumococcal vaccination should be performed before initiation of MTX.

## Abbreviations

ARR: Antibody response ratio
DMARD: Disease modifying anti-rheumatic drug
ELISA: Enzyme-linked immunosorbent assay
GMC: Geometric mean antibody concentration
IPD: Invasive pneumococcal disease
MTX: Methotrexate
OPA: Opsonophagocytic activity
PCV13: 13-valent pneumococcal conjugate vaccine
PPV23: 23-valent pneumococcal polysaccharide vaccine
pSS: Primary Sjögren’s syndrome
RA: Rheumatoid arthritis

## References

[1] Naz SM, Symmons DPM (2007). Mortality in established rheumatoid arthritis. Best Pract Res Clin Rheumatol.

[2] Flament T, Bigot A, Chaigne B, Henique H, Diot E, Marchand-Adam S (2016). Pulmonary manifestations of Sjögren’s syndrome. Eur Respir Rev.

[3] Doran MF, Crowson CS, Pond GR, O’Fallon WM, Gabriel SE (2002). Frequency of infection in patients with rheumatoid arthritis compared with controls: a population-based study. Arthritis Rheum.

[4] Doran MF, Crowson CS, Pond GR, O’Fallon WM, Gabriel SE (2002). Predictors of infection in rheumatoid arthritis. Arthritis Rheum.

[5] Singh JA, Cameron C, Noorbaloochi S, Cullis T, Tucker M, Christensen R (2015). Risk of serious infection in biological treatment of patients with rheumatoid arthritis: a systematic review and meta-analysis. Lancet.

[6] Wotton CJ, Goldacre MJ (2012). Risk of invasive pneumococcal disease in people admitted to hospital with selected immune-mediated diseases: record linkage cohort analyses. J Epidemiol Community Health.

[7] Kapetanovic MC, Saxne T, Sjöholm A, Truedsson L, Jönsson G, Geborek P (2006). Influence of methotrexate, TNF blockers and prednisolone on antibody responses to pneumococcal polysaccharide vaccine in patients with rheumatoid arthritis. Rheumatology (Oxford).

[8] Kapetanovic MC, Roseman C, Jönsson G, Truedsson L, Saxne T, Geborek P (2011). Antibody response is reduced following vaccination with 7-valent conjugate pneumococcal vaccine in adult methotrexate-treated patients with established arthritis, but not those treated with tumor necrosis factor inhibitors. Arthritis Rheum.

[9] Centers for Disease Control and Prevention (CDC) (2012). Use of 13-valent pneumococcal conjugate vaccine and 23-valent pneumococcal polysaccharide vaccine for adults with immunocompromising conditions: recommendations of the advisory committee on immunization practices (ACIP). MMWR Morb Mortal Wkly Rep.

[10] Aletaha D, Neogi T, Silman AJ, Funovits J, Felson DT, Bingham CO (2010). 2010 Rheumatoid arthritis classification criteria: an American College of Rheumatology/European league against rheumatism collaborative initiative. Arthritis Rheum.

[11] Shiboski CH, Shiboski SC, Seror R, Criswell LA, Labetoulle M, Lietman TM (2017). 2016 American College of Rheumatology/European league against rheumatism classification criteria for primary Sjögren’s syndrome: a consensus and data-driven methodology involving three international patient cohorts. Ann Rheum Dis.

[12] Online GCP. Guideline for Good Clinical Practice. Available from: http://www.onlinegcp.org. Cited 28 Feb 2017.

[13] World Health Organization. The WHO consensus pneumococcal IgG ELISA. Training manual for enzyme-linked immunosorbent assay for the quantitation of Streptococcus pneumonia serotype specific IgG (Pn PS ELISA): a guide to procedures for qualification of materials and analysis of assay performance. Available from: https://www.vaccine.uab.edu/uploads/mdocs/ELISAProtocol(007sp).pdf. Cited 9 May 2017.

[14] Nived P, Nagel J, Saxne T, Geborek P, Jönsson G, Skattum L (2017). Immune response to pneumococcal conjugate vaccine in patients with systemic vasculitis receiving standard of care therapy. Vaccine.

[15] Martinez JE, Romero-Steiner S, Pilishvili T, Barnard S, Schinsky J, Goldblatt D (1999). A flow cytometric opsonophagocytic assay for measurement of functional antibodies elicited after vaccination with the 23-valent pneumococcal polysaccharide vaccine. Clin Diagn Lab Immunol.

[16] Orange JS, Ballow M, Stiehm ER, Ballas ZK, Chinen J, De La Morena M (2012). Use and interpretation of diagnostic vaccination in primary immunodeficiency: a working group report of the basic and clinical immunology interest section of the American Academy of Allergy, Asthma & Immunology. J Allergy Clin Immunol.

[17] Hua C, Barnetche T, Combe B, Morel J (2014). Effect of methotrexate, anti-tumor necrosis factor α, and rituximab on the immune response to influenza and pneumococcal vaccines in patients with rheumatoid arthritis: a systematic review and meta-analysis. Arthritis Care Res (Hoboken).

[18] Chang Y-S, Liu C-J, Ou S-M, Hu Y-W, Chen T-J, Lee H-T (2014). Tuberculosis infection in primary Sjögren’s syndrome: a nationwide population-based study. Clin Rheumatol.

[19] Nived P, Jørgensen CS, Settergren B (2015). Vaccination status and immune response to 13-valent pneumococcal conjugate vaccine in asplenic individuals. Vaccine.

[20] European Medicines Agency. Prevenar 13: Summary of product characteristics. Available from: http://www.ema.europa.eu/docs/en_GB/document_library/EPAR_-_Product_Information/human/001104/WC500057247.pdf. Cited 8 May 2017.

[21] Romero-Steiner S, Frasch CE, Carlone G, Fleck RA, Goldblatt D, Nahm MH (2006). Use of opsonophagocytosis for serological evaluation of pneumococcal vaccines. Clin Vaccine Immunol.

[22] Brauner S, Folkersen L, Kvarnström M, Meisgen S, Petersen S, Franzén-Malmros M, et al. H1N1 vaccination in Sjögren’s syndrome triggers polyclonal B cell activation and promotes autoantibody production. Ann Rheum Dis. 2017;76(10):1755–63. doi:10.1136/annrheumdis-2016-210509 10.1136/annrheumdis-2016-210509 PMC5629946 28760805

